# Case report on the successful removal of an organic penetrating object into the orbit

**DOI:** 10.5249/jivr.v6i1.323

**Published:** 2014-01

**Authors:** Leyla Rezae, Farid Najafi, Mehdi Moradinazar, Tooraj Ahmadijouybari

**Affiliations:** ^*a*^Imam Khomeini Hospital, Kermanshah University of Medical Sciences, Kermanshah, Iran.; ^*b*^Department of Epidemiology, School of Public Health, Kermanshah University of Medical Sciences, Kermanshah, Iran.

**Keywords:** Orbit penetrating object, Case report, Surgery

## Abstract

The penetration of objects into the orbit can lead to blindness and even to the death of the patient. The penetration of organic objects longer than 7cm into the eye is a rare phenomenon. In this study, we report a case in which a 6-year-old boy fell on a pencil which penetrated the upper side of his right eye orbit. Because of the agitation of the child and the lack of access, it was not possible to perform a brain or orbital computed tomography (CT) scan, but an X-ray showed that the object had gone directly into the retro-orbital space. As the result of a clinical diagnosis, it was possible to ascertain that the globe was severely hypertonic. Throughout this process the child was extremely agitated. After consultation with the neurosurgery service, the patient was rushed to the operation room. After anesthesia and superanasal peritomy, the pencil was removed slowly from the orbit. Neurology and CT scans after surgery didn’t show any ocular or brain symptoms. Once the patient’s general condition had improved sufficiently and his visual acuity had returned to 10/10, he was discharged from the hospital. This case shows that even without specialized tests, such as CT scans, an organ can be saved.

## Introduction

Trauma or penetration of a foreign object into the eye is one of the most common causes of monocular blindness.^1,2^ Ocular trauma is the cause of blindness in approximately half a million people worldwide.^3,4^ Only one quarter of all eye traumas lead to permanent or temporary loss of vision.^4^ Thirty to fifty percent of all ocular traumas are due to orbital trauma. These injuries mostly lead to sight loss or death.^2^ Penetrating objects can be classified according to their composition into a) metallic, such as steel and iron; b) nonmetallic and inorganic, such as glass and plastic; and c) organic, such as wood or vegetable matter.^5^

The penetration of foreign objects longer than 7cm into the eye is exceptionally rare and even rarer is the removal of an object of this size without brain or ocular damage.^1^

## Case Study

**Figure 1:**
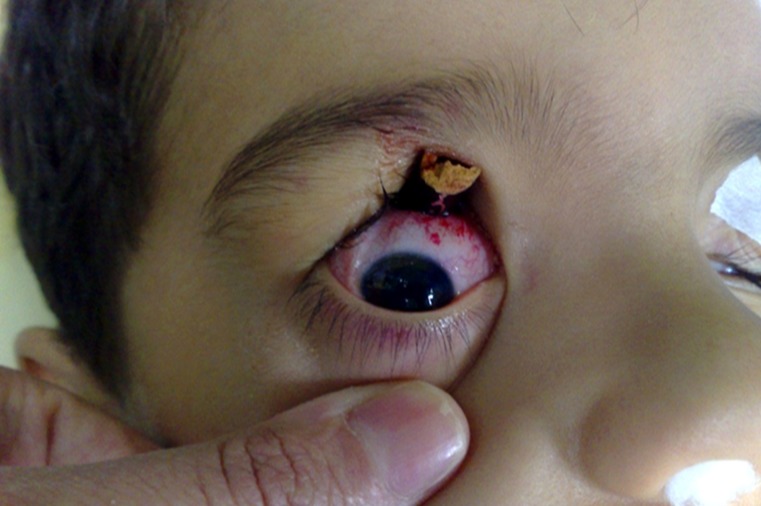
The penetration of foreign object into the upper cavity of right orbit

In 2011, a 6-year-old boy with orbital trauma in the form of a foreign object having penetrated his right eye attended the Ophthalmology Department of Imam Khomeini Hospital in Kermanshah. According to his parents, he suddenly fell when playing and a pencil went directly into the upper part of his right orbit. The severity of the accident was such that the pencil was split in two, with half of it lodged in the upper cavity of his right orbit. Because the pencil had broken at the entry location, it was under no circumstances to be moved (Figure 1).

**Figure 2:**
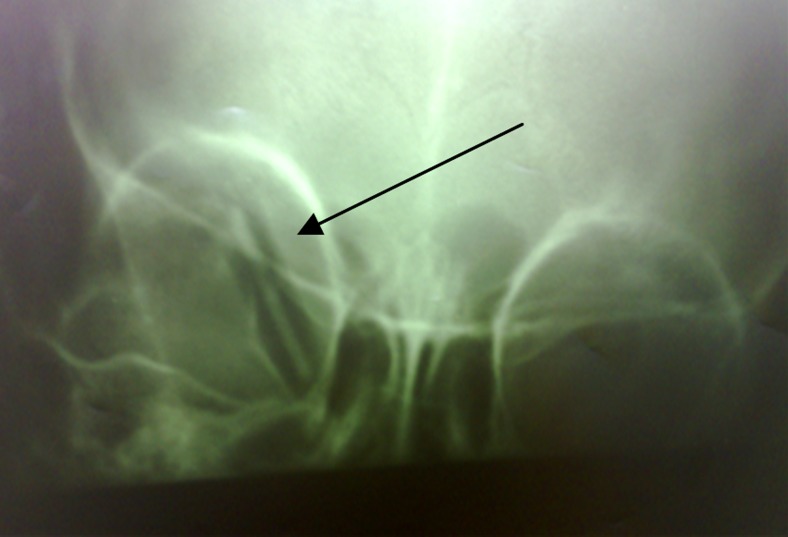
X-ray of the existence of an object with low density in retro-orbital space

A physical examination by slit lamp biomicroscope showed no damage to the boy’s cornea. The conjunctiva in the upper part of the eye had small lacerations and there was sub-conjuctival hemorrhaging at the entry point of the pencil. The globe was severely hypertonic and tense. There were no other abnormal signs in the posterior and anterior segment of the eye. As the child was in great pain and severely agitated and because of the lack of immediate access, it was impossible to do a computed tomography (CT) scan. However, X-ray (Figure 2) showed a straight, hypodense object similar to the wood density of a pencil and with a hyperdense center consistent with the carbon density of a pencil at an indefinite depth in the retro-orbital space.

**Figure 3:**
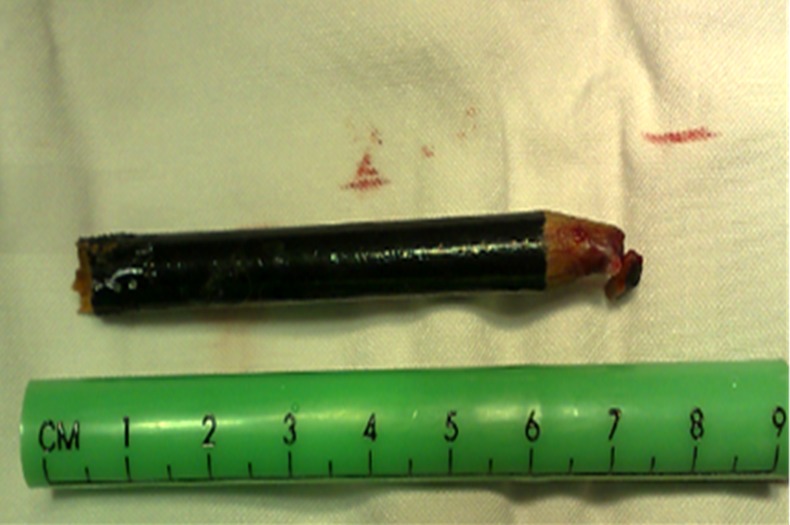
The removed pencil from orbit space

**Figure 4:**
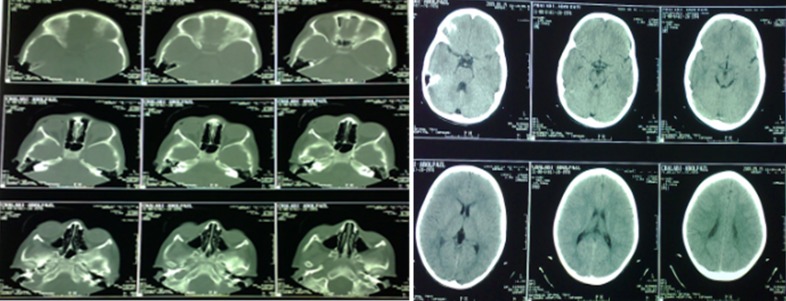
Post-surgery CT-scan

The patient was rushed to the operation room to try to save his eye. After general anesthesia and some superior conjunctival peritomy to the upper part of the conjunctiva at the entry point, the pencil was taken out of the orbit. The pencil was 7cm long ( Figure 3). By comparing the two broken sections of the pencil, we could confirm its complete removal. To be absolutely sure, a CT scan was performed (Figure 4).

The CT-scan showed the complete removal of the foreign object from the orbit and no ocular or brain damage. The patient was supervised for two months after surgery and showed no adverse effects from the trauma. The visual acuity of the patient was 10/10.

## Discussion

One of the reasons this case is of special interest is because the penetrating object consisted of organic matter. The penetration of such objects into the orbit is rare compared to that of metal and non-metal objects. These types of objects are poorly tolerated, elicit an intense inflammatory reaction, and need to be removed urgently. During the removal of objects, and especially organic objects, a number of points should be considered. Because of the low density of these foreign objects, they can be difficult to spot on a CT scan, particularly when they have a diameter of less than 0.5mm.^5^ If these objects remain inside the orbit, they may disintegrate and thus create problems for the patient. If, as in this case, the object has broken off at the entry point, it is vital that the two sections be compared to ensure complete removal of the penetrating section. If the penetrating object is a pencil, the specialists should consider the removal of the broken tip of the pencil.

Another interesting aspect of the case was that, due to the child’s agitated state, it was not possible to perform a CT scan as a diagnosis measurement prior to treatment. A CT scan would have played an important role and shown properties such as bone fracture and soft tissue damage, as well as indicating the nature and localization of the foreign object inside the orbit.^1,6,7^ However, if a CT-scan is not possible because of an inability to position the patient properly and gain sufficient access to the trauma area (due, as stated previously, to the severe agitation of the young patient), the patient can, after neurosurgery consultation, be transferred to the operation room without additional tests to save an organ. Thus, first a detailed history of the trauma should be taken from the patient. Any negligence in obtaining an accurate history of the trauma can lead to a false diagnosis and improper treatment of the patient.^8^ Although a CT scan can help identify the penetration damage to the eye to a sensitivity of 75% and a specificity of 95%, it is no substitute for direct observation and clinical diagnosis.^9,10^
